# Delivering the Multisensory Experience of Dining-Out, for Those Dining-In, During the Covid Pandemic

**DOI:** 10.3389/fpsyg.2021.683569

**Published:** 2021-07-21

**Authors:** Charles Spence, Jozef Youssef, Carmel A. Levitan

**Affiliations:** ^1^Department of Experimental Psychology, Oxford University, Oxford, United Kingdom; ^2^Chef/Patron, Kitchen Theory, London, United Kingdom; ^3^Department of Cognitive Science, Occidental College, Los Angeles, CA, United States

**Keywords:** takeaway, COVID-19, dining-in, dining-out, fine dining, gastrophysics

## Abstract

In many parts of the world, restaurants have been forced to close in unprecedented numbers during the various Covid-19 pandemic lockdowns that have paralyzed the hospitality industry globally. This highly-challenging operating environment has led to a rapid expansion in the number of high-end restaurants offering take-away food, or home-delivery meal kits, simply in order to survive. While the market for the home delivery of food was already expanding rapidly prior to the emergence of the Covid pandemic, the explosive recent growth seen in this sector has thrown up some intriguing issues and challenges. For instance, concerns have been raised over where many of the meals that are being delivered are being prepared, given the rise of so-called “dark kitchens.” Furthermore, figuring out which elements of the high-end, fine-dining experience, and of the increasingly-popular multisensory experiential dining, can be captured by those diners who may be eating and drinking in the comfort of their own homes represents an intriguing challenge for the emerging field of gastrophysics research; one that the chefs, restaurateurs, restaurant groups, and even the food delivery companies concerned are only just beginning to get to grips with. By analyzing a number of the high-end fine-dining home food delivery options that have been offered (in the UK and in the US) in this narrative review, we highlight a number of promising directions for those wanting to optimize the at-home multisensory dining experience, wherever in the world they might be.

## Introduction

The home food delivery business has been expanding rapidly in recent years, spanning everything from the delivery of takeaway meals, through part-prepared meals to boxes of raw ingredients (Moore, 2017; Kang and Haddon, 2020; Pearson-Jones, 2021). In no time at all, or so it would seem, brands such as Just Eat and Deliveroo, have become household names in those countries where they have successfully managed to establish a presence (Feehan, 2021). Over the last few years, in the UK market in particular, Just Eat, Domino's Pizza, and RooFoods (parent company of Deliveroo) were the three largest players by turnover (see Lock, 2020). Other companies such as Blue Apron, Gousto, UberEats, Hello Fresh, Serious Eats, GrubHub, Swiggy, Postmates, DoorDash, foodpanda, Zomato, etc., have also managed to develop a successful foothold in many of the countries in which they operate.

One important distinction to highlight at the outset is between takeaway meals and food boxes. Takeaway food is fully prepared and ready to eat on arrival (save for perhaps, warming up in the microwave, stove, or oven, and plating on your own crockery)^1^. McDonald's and KFC have both jumped on the home delivery bandwagon (Evans, 2017). Boxes, or meal kits (Anon, 2021), on the other hand, imply that the food has only partially been prepared, meaning that the customer is also involved in the process of preparing/finishing the meal. This may potentially result in them feeling a part of the process of making, which might itself be expected to convey certain benefits in terms of the latter's enjoyment of the food (Dohle et al., 2014; Spence, 2017a). Many fine dining restaurants have opted for the latter option (i.e., meal kits), given that high-end cuisine typically does not travel well, nor is it likely to be well-presented on delivery (which is, of course, often what makes the difference with high-end dishes; Elliott, 2015; see Spence et al., 2016, for a review).

**Figure 1:**
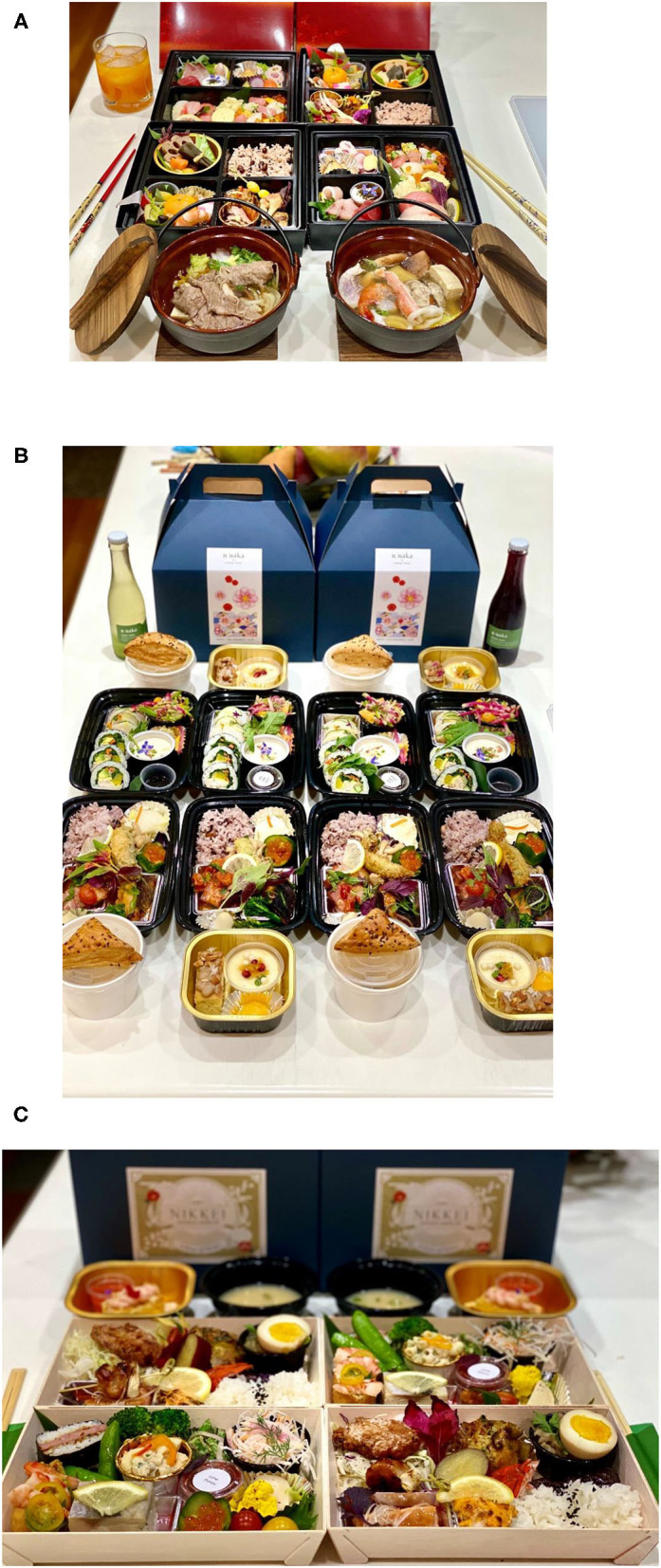
**(A)** n/naka's New Year's Eve hot pot + bento box; **(B)** n/naka's collaboration with Susan Yoon; (**C)** n/soto bento box.

As a case in point, consider only the difficulty of trying to preserve/recreate some of the beautiful dishes served at n-naka in California, at home (see https://n-naka.com/). The challenge perhaps explaining why the restaurant pivoted to bento boxes instead (see Figure 1A). Bento (弁当), which is very popular in Japan, describes a single-portion home-packed or take-out meal. The way fine dining dishes are designed (with freshness in mind, never forgetting the concept of service á* point*) makes delivering these foods more challenging, and also means the food is less likely to travel well. Meanwhile, world-famous Michelin-starred modernist restaurant Alinea, in Chicago, known for dishes such as its edible green apple balloon (Forbes, 2012), would presumably be very difficult, if not impossible, to transport to the home environment. Intriguingly, Alinea now offers their patrons the opportunity to make a number of their famous table-top desserts (see Spence and Piqueras-Fiszman, 2014) at home. What is more, social media also increasingly allows for the possibly of showing off one's personal creations online too (Olsen, 2020a).

At the same time, however, it is also worth noting how the majority of consumers have been spending much more time at home during lockdown (Spence, 2020c), and home-cooking/baking has become noticeably more popular (e.g., see The National Trust, 2020). For instance, according to Kraterou (2020), half a billion more meals were cooked at home during the first 6 months of lockdown in the UK alone. What is more, there would also appear to be growing interest from many consumers in learning from chefs with cooking classes etc., held over Zoom or some other internet platform. The Covid-19 pandemic has undoubtedly also changed the face of fine dining in many countries, such as for example, in the UK (where the majority of restaurants were closed for many months; see Clay, 2021), and in the US, where many cities have had periods of only allowing very limited outdoor dining.

Perhaps unsurprisingly, the hospitality sector is in freefall in many parts of the world. To give some sense of the scale of the problem, according to a survey from the National Restaurant Association, more than 110,000 restaurants in the US, or one in every six, had either closed permanently, or else on a long-term basis, during the first 6 months of the pandemic (see Gonzalez, 2020; Singh and Gonzalez, 2020). Given such a challenging operating environment, many of those restaurants and restaurant groups that are still in operation have pivoted to offering food for home delivery or takeaway. This raises a number of important questions about how to recreate the restaurant experience at home, which is something that a growing number of consumers apparently crave (Kraterou, 2020). Here, it is worth noting that the challenges associated with recreating the experience of dining-out amongst those forced to dine-in during the Covid-19 pandemic is presumably just going to be that much harder for those trying to deliver a high-end, or immersive, experiential meal than for those offering mainstream takeaway food (e.g., burgers, pizza, etc.).

## On The Growing Popularity of Home Food Delivery

First, though, before taking a closer look at the high-end food delivery market, it is perhaps worth briefly summarizing some of the dramatic changes that have been documented over the last 5–10 years in the takeaway sector. According to The Takeaway Economy Report (2015), in the UK in 2014, 1.2% of the total weekly spend was already going on takeaway meals, with Just Eat processing 45.5 million food orders that year. The takeaway sector was worth an estimated £9 billion to the UK economy in 2014, representing a 25% increase since 2009 (when the sector was valued at £7.2 billion). The estimated 35,000 takeaway restaurants that were operating in the UK in 2014 were already supporting a wide variety of different cuisines (especially in urban centers), including everything from South African to Mongolian, Peri Peri to Polish, and from Kurdish to Iranian cuisine. Long gone, in other words, are the days (e.g., in the 1970s) when more than 70% of all takeaway spending in the UK was on fish and chips (The Takeaway Economy Report, 2015)^2^. By 2014, that figure had dropped to just 30%, and was continuing to decline. While part of this change in the public's tastes can simply be put down to a growth of interest in more exotic takeaway fayre (The Takeaway Economy Report, 2015), the steadily increasing price of fresh fish, and the growing perception of what was once the UK's most popular takeaway food, as being unhealthy have undoubtedly also contributed to its declining popularity (see Robineau, 2016; Timmins, 2017).

**Figure 2:**
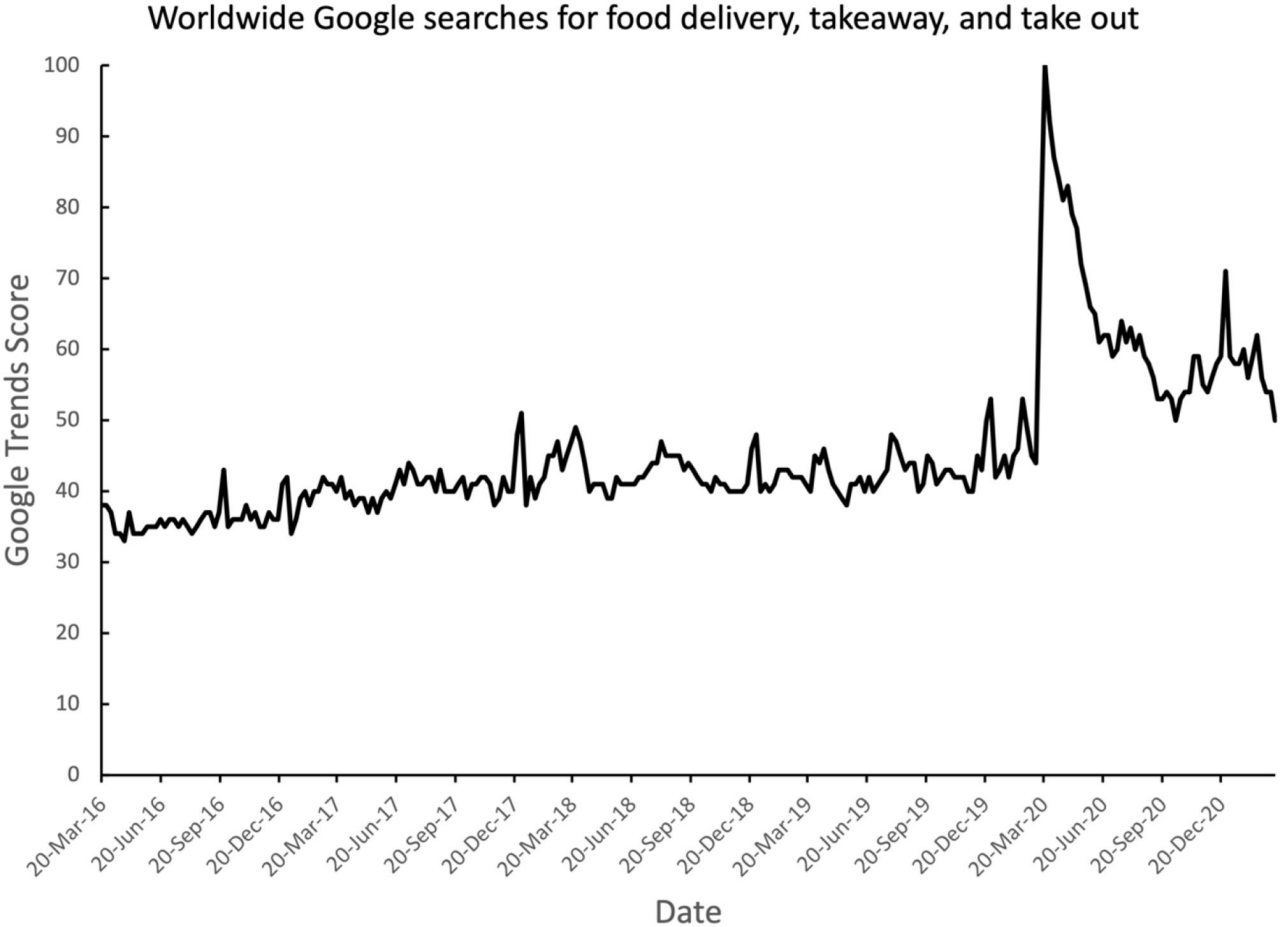
Google Trends data highlighting the major increase in combined online searches for “food delivery,” “takeaway,” “take away,” “takeout,” and “take out” in spring 2020, shortly after the global Covid pandemic lockdowns took hold around the globe. Data are scaled such that a score of 100 represents the peak number of searches.

Perhaps the most important change in the takeaway market over the last 5–10 years or so, though, has been the rise of home delivery services. These companies promise to connect the consumer (i.e., the home diner) with a range of takeaway options. A host of new ordering platforms have come online over the last decade, such as UberEats, Caviar, Postmates, and DoorDash. Indeed, according to a blogpost by Allen (2016), these four platforms alone were already processing $400 million orders (in the US) in 2014, with that figure expected to quadruple to $1.6 billion by 2016. Allen highlights how the problem for many independent restaurants is their lack of a dedicated mobile app and/or a dedicated online ordering platform. The danger is that the deleterious consequences of this digital neglect are likely to become increasingly apparent, given Allen's (2016) further claim that: “*Digital online ordering is growing 300% faster than dine-in ordering.”* The recent spike in online searches for home food delivery and takeaway certainly supports the explosion of consumer interest resulting from the Covid pandemic (see Figure 2). To give some sense of the history here, ordering food online is thought to have started in Northern California in 1995 and later expanded throughout California. The company was called World Wide Waiter and is now known as Waiter.com. The market for mobile food ordering on smartphones via smartphone and mobile apps was estimated to become a $38 billion industry and make up nearly 11% of all quick-service restaurant sales by 2020 (Wasilefsky, 2017).

With the pandemic, therefore, the use of food delivery apps has surged, bringing in billions in revenue. That said, profitability in the sector remains elusive (Sumagaysay, 2020), in part due to the rapid expansion in the major players in the area (e.g., Just Eat and Deliveroo; Witherow, 2021), as they try to dominate the market (e.g., in Europe), thus meaning that in the UK such companies that expand rapidly while posting annual losses do not have to pay any corporation tax (Walters, 2020). In certain cases, these companies also receive substantial digital enterprise grants from the UK government (Walters, 2020). Bear in mind here only that Deliveroo is backed to tune of £500 million by Amazon (Walters, 2020)^3^.

Some restaurants ship food across the US; currently for example, one can order the famous turtle soup from New Orleans' Commander's Palace either alone or as part of a multi-course dinner with spiced sugarcane quail and pecan pie, or signature dishes from New York City's Momofuko, such as their Bo Ssäm or Ko Foie foie gras (both available via https://www.goldbelly.com/). However, assembly may be required; while some dishes only require reheating, others involve some pretty extensive preparation on the part of the consumer.

### On the Rise of the “Dark Kitchen”

The phenomenal growth in the popularity of home food delivery, facilitated by the rise of online platforms, has led to increasingly vocal concerns being raised around the rise of so-called “dark kitchens” (e.g., Butler, 2017a,b; Walters, 2020), also known as “ghost kitchens” (cf. Robertson, 2013; Isaac and Yaffe-Bellany, 2019). For instance, in the US, a single restaurant might serve dishes associated with as many as 10 different brands (Wiener, 2020). Oftentimes, the food offerings are highly specialized (e.g., breakfast burritos, specific styles of pizza, or grilled cheese) in order to draw in those customers with a specific craving, and the concepts are designed to optimize visibility in the relevant food apps (Conrad, 2021). Even some fine dining restaurants have gotten on board (Krader, 2021), while some celebrities have now even formed their own “franchises,” where different kitchens prepare meals to particular specifications (Conrad, 2021).

In the UK, meanwhile, commentators have started to draw attention to the fact that an increasing percentage of branded takeaway food offerings are now being prepared not in a branch of the namesake restaurant (as the consumer might expect), but rather in one of Deliveroo's dark kitchens (otherwise known as Rooboxes). A large number of such typically-windowless shipping containers, or other temporary buildings, have been placed in anonymous industrial estates or car-park across the UK (e.g., Butler, 2017a,b; Walters, 2020). Deliveroo makes meals for famous brand name restaurants such as MEATLiquor, Busaba Eathai, Indian chain Dishoom, popular burger joints, Honest Burger, Shake Shack, and Patty And Bun, and curry house Moto. These are then delivered from Roobox outlets. Some more exclusive/expensive, restaurants such as Hakkasan in Mayfair, London have also started to offer high-end Chinese food at home as a result of Covid, including their signature Chinese dishes such as Peking duck (£110) and roasted silver cod in champagne and honey for £52. Yet much of the food in this case turns out to be prepared in a dark kitchen (Walters, 2020)^4^. Given that such dark kitchens often bypass planning regulations and give rise to noise disturbance from all of the delivery vans and mopeds coming and going to those living nearby, Deliveroo has been facing a growing chorus of complaints from local authorities/councils in the UK (e.g., Butler, 2017a).

While it presumably should not really matter where exactly one's meal is made (providing, that is, that the end result tastes as expected), one might nevertheless want to question whether it is still worth paying a premium for the “brand,” especially for what might essentially be considered a commodity takeaway item such as a burger, say. At the same time, however, it is interesting to note that the question of where exactly it was made never seems to arise in the mind of the consumer when enjoying processed foods (cf. Laudan, 2001). So what, exactly, is different in the case of branded takeaway meals dispatched from dark kitchens rather than the namesake restaurant? Of course, it is a separate question as to whether this should, or does, change, the consumer's experience of a meal, and, if so, how. At the same time, however, concerns about the source, or location, of food preparation do not seem to have been raised in the context of fine-dining. Perhaps this is because the quality of the food is supposed to speak for itself. What is more, diners often have to pick up the meal kit from the restaurant itself, rather than having it delivered to the home, thus reinforcing the notion that the food was actually prepared there^5^.

### High-End Meals At-Home

Launched in 2015, Supper (https://supper.london/home) has been delivering Michelin-starred restaurant cuisine direct to the door of those living in central London for more than 5 years now (e.g., Editorial Staff, 2015; Anon, 2020). According to a recent press report (Vincent, 2020), they currently service customers in the Home Counties as well (i.e., the range of delivery has expanded over the years). In line with what we have seen so far in this review, the company reported a 700% increase in orders since the start of lockdown. The company apparently has some clients who regularly pay £1,500 for Michelin-starred restaurant meals to be brought from London (Vincent, 2020). The latter example, assuming that it is more than just an isolated instance, could then be taken to support the claim that the most expensive home-delivery option may constitute a successful business opportunity for high-end restaurants (Vincent, 2020), that is, a high-end meal offering with a financially-viable take-up amongst consumers. Supporting such a suggestion, Deliveroo also reports that it has been delivering some increasingly expensive meals to its customers (Scott, 2017).

To those of us who may be unaccustomed to paying such astronomically high prices for a meal, never mind a takeaway, or meal kit, one might be tempted to wonder whether such experiences represent a one-off indulgence, or else a genuine and sustainable shift in dining patterns. The alternative here is to consider only whether the exceptionally high prices might be promoted in order to help maintain perceived exclusivity of the food brand without the restaurant necessarily expecting much of a take-up (Wharton, 2008; Poundstone, 2010). If this sounds unlikely, consider only how pre-Covid, excessively expensive options were sometimes put on restaurant menus seemingly in order to elicit outraged press interest (at the expense) and/or to make the other options on the menu look cheaper (Spence and Piqueras-Fiszman, 2014)^6^.

However, despite this interest in the delivery of high-end food to the home, many chefs and restaurateurs have realized that it is difficult to reproduce the multisensory experience of a high-end restaurant meal at-home. It certainly requires far more than simply just ordering the suitably-expensive home-delivery meal kit direct to one's door, or else picking it up from the restaurant itself. The question to be addressed in the remainder of this review, therefore, is how chefs, restaurateurs, and restaurant groups have adapted in light of the dramatic changes in behavior that have been brought about by COVID. We also highlight a number of promising directions for those wanting to deliver an enhanced multisensory dining experience at home that goes well beyond merely providing the food (and drink) itself.

## Is it Possible to Deliver a High-Value, or Experiential, Meal at Home?

**Figure 3:**
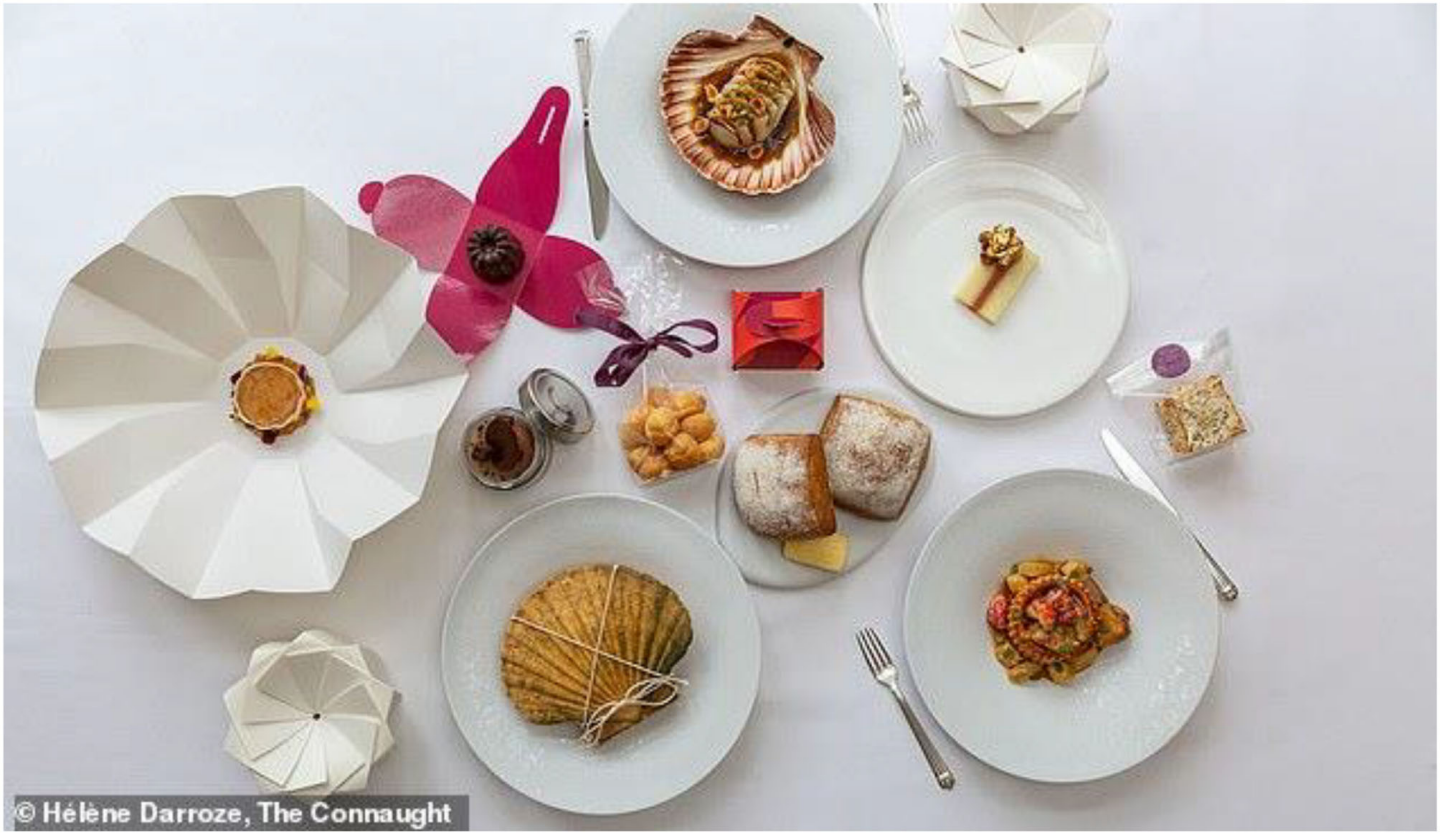
£415 takeaway meal for two from Hélène Darroze at The Connaught Hotel, London (McCarthy, 2020). Figure copyright Hélène Darroze, The Connaught.

The expectations of takeaway/fast food are typically not that high (Cowen, 2012). What is more, takeaway meals likely do not deteriorate much in transit. In fact, it has even been suggested that the flavor or certain takeaway meals improves on reheating (see Pass Notes, 2020). By contrast, one might wonder whether it is actually possible to deliver a high-value meal, or even one of the increasingly-popular immersive (or experiential) meals, for those dining at home. For example, just take the five-course New Year's takeaway menu from high-end London hotel The Connaught in Mayfair that cost £415. The meal, in this case, was prepared by Michelin-starred chef Hélène Darroze (McCarthy, 2020), with diners presented with a number of distinctive features/elements, including beautiful origami packaging (see Figure 3), a point to which we will return later. But can the culinary experience at-home ever really justify the expense (or live up to the experience of dining-out)?

Many other high-end restaurants in the UK and US have also jumped on the meal kit bandwagon since the start of Covid. They include Alinea in Chicago (one of the first to do so—and at a reasonable price; Olsen, 2020b); and, in the UK, Restaurant Hyde in London (https://hydeandco-delivery.co.uk), Core by Clare Smyth (https://www.corebyclaresmyth.com/core-at-home/), La Gavroche (https://www.hot-dinners.com/2020111110026/Gastroblog/Latest-news/michel-roux-jr-jason-atherton-restaurant-box-le-gavroche), Sketch, Simon Rogan and many, many others (https://luxurylondon.co.uk/taste/food/home-deliveries-restaurants-london; e.g., Anon, 2020; Chomka, 2020; West and Henderson, 2020). While some restaurants have opted for ongoing offerings, with regular menu changes, others only offer menus for special occasions.

While the focus of this narrative review is squarely on high-end dining in the UK/US, it is worth noting that similar trends have been reported in a number of other countries including Australia (Amin, 2020; Wilden, 2020), Canada (Bell, 2020), and Japan (Kyodo, 2020).

However, can an expensive takeaway meal or meal kit ever really come close to a delivering what one expects from a high-end experience when dining-out if consumed in the customer's own home? After all, the atmosphere etc. is an important factor influencing the dining experience (e.g., see Anon, 1965; Spence and Piqueras-Fiszman, 2014; Spence, 2020a). Indeed, legendary French chef Paul Bocuse is once rumored to have said that more than half the experience of fine dining is comprised of “the everything else” (i.e., beyond the food and drink offering itself; see Spence and Piqueras-Fiszman, 2014). And while one might well be tempted to question what the appropriate percentage should be in this case, there can be little doubting that the service element is critical to the customers' experience of fine dining (e.g., Matthews, 2017)^7^. One might also wonder how important the music is (Wilson, 2003; Fiegel et al., 2014; Spence et al., 2019b), or the scent, the flower arrangements, the napkins, or even the lighting (see Spence, 2021a, for a review)?

In a way, the appreciation of food, in particular, high-end cuisine is much like art, being rated more highly when experienced in the appropriate context (see Brieber et al., 2015). Here, it is interesting to note how the same meal is sometimes rated very differently by participants depending on where it happens to be served—five-star hotel restaurant or institutional cafeteria, for instance (e.g., Bell et al., 1994; Edwards et al., 2003). In such cases, however, it is important to stress that the location/venue may have been the only product-extrinsic cue that the diners had to go on when it came to rating the quality of the food. Contrast this with the situation when eating an expensive meal kit at home. In the latter case, the diner is presumably acutely aware of the brand that is supposedly providing the food and hence the atmosphere/location might be expected to matter less (i.e., branding matters, while the means by which that branding information is conveyed, does not).

## The COVID Pandemic and the Changing Face of High-End Hospitality

According to the many articles that have appeared in the UK press, there have been a number of sometimes dramatic changes in the foods that people are choosing to buy/eat during Covid lockdown (e.g., Hargreaves, 2020; Pearson-Jones and Poulter, 2020). Changes have been reported both in terms of what people choose to consume and how we choose to prepare it (if at all). Home cooking/baking was also much more evident in the initial periods of lockdown than has been the case in the later ones (The National Trust, 2020). According to one report in the British press, for instance, sales of trifle were up by 700%. While there was marked panic buying (e.g., of pasta) during the initial periods of lockdown (e.g., in the UK, see Bekiempis, 2020; Lufkin, 2020)^8^, it is worth stressing that somewhat different patterns of consumer behavior have been documented during each period of lockdown. Note here also that tension and stress (e.g., associated with Covid-related stresses and strains) have been shown to lead toward greater desire for, and consumption of, fast food and sweet/carbohydrate-dense foods (Leow et al., 2020). There would also seem to be an increasing use of food rituals amongst consumers to help deal with Covid-related stress (Randall, 2021; Wang et al., 2021a), as well as a rise in consumption of nostalgia foods (Morrissy Swan, 2020; see also Cereceda, 2020). Here, though, it is worth noting that often what is called a food ritual turns out, on closer inspection to be more of a food habit, or stereotypical food behavior, meaning that it lacks the traditional/symbolic element of true rituals (see Spence, inpress; Visser, 1991).

But have our tastes/preferences as far as high-end, or immersive experiential dining, also been changed as a result of Covid? Furthermore, there is a very real question here as to whether diners will still be interested in experiential/molecular/modernist dining in the post-Covid era (see Spence and Youssef, 2018; Spence, 2020c)^9^. Here, though, it should be noted that it is currently difficult (i.e., during Covid) to predict which of the Covid-related trends in food and drink consumption will stay post lockdown, and which may revert to their former patterns of food behavior/preference (Plata et al., submitted). Intriguingly here, Some restaurants have been encouraging their former customers to buy vouchers for future dining-out meals in anticipation of when the hospitality industry opens up (Lutrario, 2020).

### Changing High-End Food Offerings

Intriguingly, the chefs in a number of the most famous restaurants, have chosen to adapt their menus and pricing to be much more accessible during Covid. There has also been a change in what is served in many venues. For example, Noma, in Copenhagen, formerly judged to be the world's best restaurant, served what can perhaps best be described as experimental Nordic cuisine prior to the Covid pandemic. However, on reopening in May, 2020, the offering consisted of a much more homely combination of burgers and wine/beer (Hosie, 2020). These items can be enjoyed in the restaurant's garden or else be taken-away for only $18. Note that this is just 1/20^th^ of the cost of the usual Noma experience (excluding drinks). Meanwhile, at another of the world's former top restaurants, The Fat Duck (in the UK) has started offering two-course lunches again after more than a decade of only serving a set menu. By contrast, at the start of the pandemic, Alinea began by offering beef Wellington, then started serving French comfort food. However, as the various periods of lockdown have progressed, it has been reported that the food has grown increasingly “Alinea-like” (Kludt, 2020).

The atmosphere is so much a part of the total experience at restaurants such as Vespertine in LA (http://vespertine.la/), from chef Jordan Kahn. A meal for two will set one back upwards of $1,000. A 5-h dinner comes with bespoke soundscape that plays pervasively throughout (from the car park through to the roof where your meal service begins and back down to the garden), scents^10^, and the most other-worldly, and beautiful plateware that the diner has likely ever seen (see Scattergood, 2017; Spence, 2020b)^11^. How, then, to offer a meal that does not compromise the essence of the in-person person experience of a meal at Vespertine? How to capture the scents, the sounds, and the ubiquitous black of the interior (not that everyone would want to; see Baum, 2017)? Much the same problem faces other (typically high-end) multisensory experiential dining concepts such as chef Jozef Youssef's Gastrophysics Chef's Table that involve everything from projection mapping on the dining table though sonic accompaniments to match certain of the dishes (Spence and Youssef, 2020; see also Pigott, 2015; Abend, 2019; for a number of other experiential multisensory immersive dining concep).

Kahn, like many other chefs wisely does not try to deliver the full restaurant experience for takeaway at home. Instead, the chef creates a completely new menu every few weeks, designed around a particular theme (Addison, 2020). While some of the themes reflect Kahn's own family heritage (Cuban, Southern US, Sicilian, and Yucatán), others are linked to his own culinary lineage (namely the French Laundry, and Alinea). Crucially, however, the price point is far different than dining in (at the restaurant), with most of the offerings starting at < $100/person.

At many restaurants, frequently changing themes/menus provide a reason for customers to return. And yet, at the same time, there is also a sense in which, there should be something constant (so that the customer has something tangible to look forward to when ordering again; see Poundstone, 2010; Spence and Piqueras-Fiszman, 2014). Limited-time themes can help to create both variety and scarcity. At David Kinch's Manresa, based in Los Gatos, California) the at-home menu changes daily (https://www.manresarestaurant.com/menus/). Kinch's well-known New American eatery offers extravagant farm-to-table tasting menus, and relevant to one of the themes that we will return to shortly, it is noticeable how the stress is on the social family meal element of the offering (just take the following from the Google search page: “MANRESA FAMILY MEAL TAKE OUT. We have released our Daily @manresafamilymeal menus for the entire week”). Given that museums, galleries, and musical venues are closed in so many major cities, and travel is heavily restricted in much of the world, food has become a way to experience art, and to at least get a taste of being somewhere else (All the more relevant when it is realized that according to a pre-Covid survey of young British holidaymakers, one in five reported that they were choosing the destination based on the food that they expected to find at their destination (Amey, 2015; see also on the desire to travel being linked to meal box delivery; Thornhill, 2021).

In some cases, there have been intriguing culinary collaborations and innovations with the move to takeaway food provision at the high-end. While n/naka (in California, this a restaurant that we came across earlier) is normally a kaiseki restaurant, they offered a bento box created by chef Susan Yoon that included Korean-inspired flavors (see Figure 1B)^12^. Given the popularity of such collaborations, the team behind n/naka ultimately decided to open a second restaurant, n/soto (see Figure 1C), specifically focused on such collaborations (Wang, 2021). In San Francisco, the chefs of Michelin-starred restaurants Lord Stanley and Mister Jiu's came together to create five-course meals (Guerrero, 2020). In other cases, restaurants have chosen to reference each other; For instance, Melisse x Citrin in Los Angeles had a series of “tribute” menus including to chefs David Kinch, Jean-Georges Vongerichten, Daniel Boulud, and Alice Waters (https://www.instagram.com/melisserestaurant/?hl=en). Meanwhile, Alinea in Chicago and Eleven Madison Park in New York City teamed up late in 2020 to collaborate on a meal offering (Krader, 2020).

## On the Economics of Fine-Dining at Home

While the consumer might well expect to pay less for a meal when delivered direct to their door (rather than served in the context of the restaurant; for instance, Cosme in New York City offers some of its food items for less on Caviar than they charge in person; see Adams et al., 2020), it is worth noting that the likes of Deliveroo demand around 35% of the cost of the meal to deliver and VAT and £3–5 delivery fee to customers (Walters, 2020; see also Merriman, 2021). That means that Deliveroo takes £42 of every £100 charged by the restaurant. By contrast, big chains, including the likes of McDonald's (Evans, 2017), Starbucks, and Wagamama, meanwhile, are charged much less (c. 20%). As such, it is really the big brands who can afford to use these dark kitchens, more than the independent restaurant^13^. Some cities have pushed back on such fees, capping them at 15% (e.g., Allyn, 2020), but this still represents a substantial hit to revenue streams. What is more, the cost of packaging pushes up the price of the at-home food delivery still further (e.g., see Mahe, 2015). Luke Johnson, the owner of Gail's Bakery as well as the former chairman of Pizza Express, had the following to say: “*I can't see how restaurants can make a profit if they have to hand over 35 per cent. And I don't really want to eat meals cooked on industrial estates that have been on the back of a moped for 20 minutes.”* (Walters, 2020). The expense may be even harder to justify to consumers given that they already appear to have an unrealistic sense of the true costs involved in preparing and serving a meal in the context of a restaurant (Peters, 2016).

There is an important question here about the economics of providing a high-end meal experience at home. Bear in mind only that many restaurateurs find that their profits derive primarily from the sale of alcohol (Cowen, 2012), whereas producing the food itself tends to be more-or-less done “at cost” (Markwell, 2017). However, if dining at home, the wily customer might rightly question why they should be expected to pay the normal 300% mark-up for a wine or drink that they can likely acquire at a fraction of the price elsewhere. Such mark-ups may all too easily strike at-home diners as excessive, and diners can Google/buy wine much cheaper (e.g., Chung, 2008; Spence and Piqueras-Fiszman, 2014, p. 56). What is more, the food/wine pairings that are such a feature of many high-end dining experiences (see Spence, 2020e, for a recent review) are obviously going to be very difficult to deliver at home, given the standard 750-ml packaging of wines.

At the same time, however, craft cocktails have been rapidly emerging in the US, with many states changing their laws in order to allow for the delivery of home cocktail. With alcohol consumption increasing during the pandemic (Pollard et al., 2020; Zipursky et al., 2021; though see also Plata et al., submitted). As such, the impact of shifting to take-away are context-specific. Even beyond alcohol sales, though, the economics of take-away are highly variable. Alinea's highest revenue day ever occurred during the pandemic—though note that one of their co-owners is also a founder of Tock, a reservations and ordering website used by many high-end restaurants, which, or so it has been argued, may have given them an edge in rapidly pivoting their business model (Kludt, 2020). It may be that some high-end restaurants will opt to continue with some take-away options even post-COVID; special holiday events, especially when they require pre-order, can allow for restaurants to reach far more customers than they could possibly accommodate in-house, and with reduced front-of-the-house staffing. Now that many people have had the opportunity to try a Michelin-starred holiday dinner at home, will they want to go back to cooking such “event meals” themselves?

## Optimizing the Experience by Delivering More Than Just Dinner

It is important to stress that the atmosphere is only a part of what makes a meal at a top restaurant so special (see Spence and Piqueras-Fiszman, 2014, for a review). Indeed, it has often been suggested that eating out is as much a social activity as anything else (see Spang, 2000; Julier, 2013; Spence, 2017a). Given the epidemic of loneliness that has resulted from lockdown (Klein, 2020), those offering food for home should really be thinking about how they can also offer hospitality^14^. For instance, Nick Kokonas, co-owner of Alinea, has talked about hospitality being “extended to the curb,” by making sure to greet the customer by name when they come to pick up their meal (Kludt, 2020)^15^. Some restaurants include thank you notes in appreciation of support during difficult times.

### Facilitating Digital Commensality

Given the growing social isolation, even before Covid struck (Spence, 2017a), it would seem that there is likely to be an important opportunity for those offering high-end dining to deliver company (perhaps digitally), and not just “sushi for one,” say (see Spence et al., 2019a). After all, a number of commentators have predicted an epidemic of loneliness caused by the Coronavirus crisis (Klein, 2020)^16^. It would likely make sense to consider how the meal kit (or takeaway) can be used as a vehicle to deliver a social encounter.

As mentioned already, the pandemic and associated lockdown has seen a marked increase in solo dining. As such, there is a growing need for digital commensality (see Spence et al., 2019a, for a review). Indeed, given that dining is fundamentally a social activity, it is the shared social interaction that has been one of the most obvious casualties of the current series of lockdowns (see also Holmes et al., 2020). As such, this is what is most obviously missing currently for so many at mealtimes.

Various studies had already documented the dangers for those dining alone in terms of lowered mood, and impaired food behaviors—either consuming more (given how few foods are portioned for just one person) through to not eating enough as a result of the negative mood that can be associated with loneliness (see Spence, 2017b). In response to this enforced (and, by now, increasingly prolonged) social isolation that so many of us have been facing, there has been a growth in digital surrogates: Everything from Zoom cocktails (of which the authors have partaken, never having done so previously; Bernard and Bastone, 2020; Smith, 2020; Tilley, 2020; see also the concept of the Quarantini cocktail, Hubbard, 2020), Zumba classes (Barr, 2020), Skeating meals online (i.e., Skyping while eating; Bernard and Bastone, 2020), which was already predicted by Spence (2017b). More people are now cooking and sharing meals together online with friends and family using platforms like Zoom or Google Hangouts (Heil, 2020). Though our sense is that drinking together online is more popular than dining together.

Note also the role that social media can play, with Steak-umm, a frozen meat company, having established a following on Twitter for their scientific literacy and critical thinking advice (Bogomoletc and Lee, 2021). Some restaurants are directly engaging with the general public on platforms such as Facebook and Instagram. Diners can now preview their meals on Instagram, seeing the photos others have taken, building a sense of anticipation, and sharing images of their food can also help to facilitate connection (though envy can also be a consequence). For many, part of the joy of creating Alinea's desserts is the aesthetic experience, as much as merely just the gustatory satisfaction (Olsen, 2020a), as each creation is unique.

In social psychology, there is emerging evidence for “enclothed cognition:” the idea that what one wears can influence one's state of mind (Adam and Galinsky, 2012). For instance, wearing a tunic identified as nursing scrubs leads to greater empathy and altruism (López-Pérez et al., 2016), while people wearing police uniforms were more likely to shoot unarmed targets in a video-game simulation (Mendoza and Parks-Stamm, 2020). Might restaurants therefore encourage at-home diners to dress for the meal? One could imagine providing an apron or toque for some of the more DIY experiences, or suggesting colors or styles for the eating experience. Relevant here, according to the latest evidence, the clothing that people wear can exert an influence on their choice of healthy food (Wang et al., 2021b). Even the color of the napkins and the presence vs. absence of tablecloths has been shown to make a difference to the experience (see Spence, 2017a, 2021a).

### Cooking Class/Advice

While restaurants simply cannot offer the at-home dining experience of being served, they can nevertheless potentially offer their guests the opportunity to become engaged in the creation, or at least the assembly, of their meals, which can lead to a greater appreciation of the food (e.g., Dohle et al., 2014; Spence, 2017a)^17^. Note that there has anyway already been growing interest in online cooking classes in recent years (e.g., Peters, 2020). For instance, Eleven Madison Park, in New York City, offered a foie-gras stuffed chicken to be roasted at home^18^ as well as a $200 “truffle and eggs” dish consisting of six raw eggs, truffles, and instructions concerning how to cook an omelet (Goldfield, 2020). Meanwhile, the Vespertine x Alinea collaboration offered diners the chance to “Be the Chef” with a dedicated hotline that patrons could call should they require some culinary assistance (Hochman, 2020). A number of other top restaurants have also created videos designed to guide the customer through the process of proper heating/assembling/plating of the food as well as to give context about the courses/the ingredients. For example, at San Francisco's Atelier Crenn, each course has its own video. One can see gloved experts using tweezers to precisely arrange the component ingredients/elements (https://ateliercrenn.getbento.com/crenn-kit-luxe-menu/; https://vimeo.com/user115409838)^19^. Los Angeles's Providence's videos all feature their chef Michael Cimarusti, and, at times, become a little playful; for instance, a menu featuring black truffles from the Southern hemisphere was accompanied by a video that opens with Men at Work's song, “Down Under” (https://www.youtube.com/watch?v=ybomgEQN7AE).

In the UK, Kitchen Theory offered a complete home multisensory experience; For instance, in February, 2021, they offered Valentine's Day boxes incorporating a four-course meal, wine, scented candle and a curated Spotify playlist (https://kitchen-theory.com/valentinesday/). In March, there was a Mother's Day box with full afternoon tea, glass cake stand, rose atomiser, luxury tea selection, and another Spotify playlist (https://kitchen-theory.com/mothers-day-afternoon-tea/). Note here how both meal boxes came with a written guide for the recipient of the meal box on how to have a more multisensory experience at home and a zoom call with the chef ahead of the experience to help them with any questions that they might have.

Hence, rather than seeing the need for the end consumer to have to do some of the preparatory work on the food in a meal kit as a disadvantage, some chefs/restaurateurs have managed to turn their interaction with their customers to their own advantage.

### Packaging, Cutlery, and Other Atmospheric Enhancements for In-Home Dining

Simply packaging high-end food in paper/cardboard or Styrofoam, like regular takeaway, and then instructing the consumer to stick it in the microwave/oven is obviously not going to set expectations of a high-end multisensory tasting experience in the mind of the customer. There is ample research demonstrating that packaging and serving/serviceware influence the meal experience (e.g., Iggers, 2007; Robinson et al., 2007; Field et al., 2009; Spence and Piqueras-Fiszman, 2014). Some restaurants have explicitly brought in multisensory aspects to the serviceware. At o.d.o. (a kaiseki speakeasy opened by Chef Hiroki Odo in New York's Flatiron District, https://www.odo.nyc/), the Valentine's Day meal came in a reusable box made of mulberry bark along with Japanese glassware and a playlist (Wiener-Bronner, 2021)^20^. Meanwhile, one of Vespertine's meals came with hand-crafted flatware made from coconut shells, handmade incense, a cedar spray, and even a selenite crystal^21^. Meanwhile, Niki Nakayama and Carole Iida-Nakayama of n/naka developed a cook-at-home meal kit that included a candle artwork and a musical playlist (Stueven, 2020).

The added extras are often what improves the offering, as in the case of the UK's Petersham Nurseries. According to one online source: “*The food is hearty and substantial, but what really elevates this particular offering is the added extras. As standard, it comes with a pair of beautiful gastro green candles to illuminate proceedings. But you can go the whole hog, with matching wine and premade cocktails*—*all the way up to a Petersham-inspired tablescape including Bertozzi tablecloths and napkins and Murano glassware.”* (Anon, 2021). Meanwhile, at one point during the evolving series of lockdowns in the UK, The Fat Duck was, offering its diners a basket of goodies to take away to complete their meals, given the Government stipulation that all venues had to be closed, or stop serving food, by 10 p.m., before banning indoor dining altogether once again (cf. https://thefatduckgrouphampers.co.uk/).

Food tastes better if consumed with heavier cutlery (Michel et al., 2015). Furthermore, given the growing interest amongst some chefs and food designers in novel forms of food interaction. Novel forms of cutlery are not unusual, such as Kitchen Theory meals served without a fork. Consider only the opening jellyfish dish that was served wrapped around tweezers as part of the Gastrophysics chef's table (Youssef et al., 2019). At the other end of the food spectrum, recognizing the importance of cutlery to the dining experience, McDonald's France have taken the decision when serving their “signature” gourmet burger (coming in at twice the price of their ordinary burger) to provide a knife and fork at all 1,400 of its French restaurants^22^. While much of the mainstream takeaway food is eaten with the hands, this approach is just much rarer in the context of high-end dining. Note here only how the provision of cutlery (e.g., in an Asian restaurant) can help to frame a meal.

In the world of product design, many companies have become aware of the power of unboxing (e.g., Kim et al., 2018). Apple, for instance, is well-known for taking great care to ensure consumers would have a positive emotional experience when opening up their new products (Lashinsky, 2012). These days there is even a YouTube genre of unboxing videos (Mowlabocus, 2020). Vespertine has managed to maintain continuity with the physical restaurant's black aesthetic by providing the food in sophisticated black boxes complete with black tissue paper that provides for a pleasurable unveiling experience (https://www.instagram.com/p/CMgJuWPJ0x-/). Meanwhile, n/naka's boxes are customized to each theme, but care is taken to ensure they are as visually appealing as possible (https://www.instagram.com/p/CExvlngBHLY/). By using higher-quality materials, restaurants can communicate the expectation that the ensuing food experience will also be a quality offering too. It is perhaps worth bearing in mind here that even the color of the napkins have been shown to affect the diner's impression/emotion (Navarro et al., 2020).

There has been growing interest at the high-end of more experiential dining to help the diner curate the multisensory atmosphere at mealtimes. This has involved everything from matching musical playlists through providing scented candles and aromatic scent sprays, and could, potentially at least, incorporate intelligent illumination recommendations too. After all, color-changing remote control lightbulbs are now cheap enough to be included, and ambient color has been shown to influence our food choice and flavor perception (see Robson, 1999; Spence et al., 2014; Cho et al., 2015; Robinson, 2016). That said, curated playlists and scent are perhaps the easiest elements to transfer from the restaurant to the home dining environment (cf. Fulberg, 2003; Guéguen and Petr, 2006).

However, by far the most widespread use of multisensory atmospheric elements, such as curated music to complement the experience of food, has been promoted by the big brands, including everyone from British Airways (Victor, 2014) through to Häagen-Dazs, and from Munchery to Just Eat. Krug champagne have also paired their Champagnes with “matching” musical playlists to help enhance the multisensory tasting experience of a high-end drink (Spence, 2017c). Meanwhile, Starbuck's Japan recently introduced the idea of simply by scanning a provided QR code, the consumers can have a sakura (cherry-blossom) latte under an AR-blooming tree (Tanquary, 2020). As such, there would seem to be great potential for home food delivery services, where the take-away, or meal, is delivered together with a curated music selection (e.g., a Spotify playlist) designed to enhance, or modify, the consumer's tasting experience: Relevant in this regard, just take Munchery and Google Play Music (e.g., Roncero-Menendez, 2015; Samuely, 2021) who teamed up to turn a simple meal into a dining experience. Twice a week from August 17^th^ through September 11^th^, 2015, as part of the daily menu offering incorporated website custom playlists paired with specific dishes (e.g., “Coffee Shop Indie Radio” paired with chocolate cake, while “Sunny Patio Vibes” paired with a lightly grilled chicken dish). Here, it is worth highlighting the fact that this more experiential approach to the delivery of takeaway food was designed to be paired with mainstream (i.e., not high end) food offering.

Having realized just how important the atmosphere is to the experience of eating and drinking (see Spence and Piqueras-Fiszman, 2014), food and drinks brands are increasingly doing everything in their power to optimize the sonic backdrop when the consumer tastes their products. It really can make all the difference (see Spence, 2020d, 2021b, for a couple of recent examples).

In a project commissioned by Just Eat in 2017, one of your authors (C.S.) and his colleagues had more than 700 people evaluate of a range of styles of food (Indian, Italian, Thai, Japanese, and Chinese) while listening to one of 19 different music tracks, designed to cover a diverse range of popular musical styles (e.g., including jazz, pop, opera, etc.). The participants were invited to estimate the spiciness of the dish, how much they thought it was worth, and how appealing it looked. Amongst a number of other findings, the results showed that jazz music resulted in the takeaway dishes being rated as significantly more expensive (a 4% lift on average). In particular, across these different styles of takeaway food, the top tracks in terms of food evaluation were Feeling Good (Nina Simone) and One for my Baby (Frank Sinatra). There is also a role for matching the music to the cuisine. Nessun Dorma sung by Pavarotti gave rise to the highest ratings for the Italian food, there would also seem to be an element of ethnic matching too. By contrast, Justin Bieber's song Baby, and the absence of music, led to the lowest ratings for the takeaway meal options viewed online. Intriguingly, classical music has often been reported to increase food and drink sales in both stores and restaurants (see Spence et al., 2019, for a review). Taken together, such results support the suggestion that taking time to intelligently select an appropriate soundtrack may help to enhance the meal experience at home—assuming, that is, that those dining at home choose to access the recommended playlists (see Sanderson, 2015; Spence, 2015). In 2015, researchers were already predicting that CDs might be thrown in with take-away delivery (see Spence, 2017c)^23^.

However, beyond simply using music to match, and hopefully enhance, the diner's experience of the meal, it might also be worth considering delivering an emotional experience. That is, it might pay off to determine whether the diners are interested in a romantic, an experiential, a comforting meal experience, and thereafter coordinating the everything else to match. Relevant here, the first author (C.S.) has also been involved in a project to coordinate home delivery of meals with top TV shows offered by Sky Atlantic (Russell, 2017), partnering with a leading chef. So, for example, this led to Deliveroo serving a Game of Thrones themed meal. Meanwhile, other shows that were airing at the time, and for which a matching meal was designed included Riviera and Twin Peaks. However, in all these cases, the short-term nature of the pairing offering suggested that the companies concerned may have been more interested in the associated press headlines (that can result from pairing the senses in unexpected ways) than necessarily with long-term improvement of their customers dining experience.

## Conclusions

It would seem that the most successful examples of high-end restaurant home food-delivery have not attempted to simply reproduce the dining-out meal experience for which they are best known in the context of dining-in. Rather they have used their brand to develop and deliver something that works given the constraints of food that will need to be finished at home (e.g., see Vespertine, Kitchen Theory Gastrophysics chef's table). Some of the more successful examples have obviously put a great deal of thought into the design of the packaging and presentation (e.g., see Vespertine). Ultimately, however, the challenge for high-end chefs, restaurants, and restaurant groups is one for gastrophysics (e.g., Robinson, 2015; Miari, 2020; Spence, 2020a). By adding more elements to the total multisensory tasting experience, such as music, scented candles, bespoke cutlery, culinary instruction, etc., it also offers the opportunity for chef to engage in a little Sensploration (Leow, 2015), which is itself becoming increasingly popular.

While the majority of examples have been taken from high-end meal box offerings, it should be remembered that several of the examples that have been discussed came from attempts to enhance regular takeaway offerings. Recognizing how lonely many people may be feeling during Covid also likely represents an important opportunity for restaurateurs. At the same time, however, it is also noticeable how the food has come down in price in many cases, and the offering has often been more oriented toward comfort foods. In closing, we should highlight the (narrow) focus of this review on UK/US fine dining scene. However, we also believe that the solutions to enhance experiential high-end dining would also be appropriate in other countries where there is an appetite for such experiential dining, and we have highlighted how the same approaches have been reported in Australia, Canada, and Japan as but three examples.

## Author Contributions

All authors listed have made a substantial, direct and intellectual contribution to the work, and approved it for publication.

## Conflict of Interest

JY is chef/patron of Kitchen Theory, a multisensory gastronomic design studio, of which author CS is also a director. The remaining author declares that the research was conducted in the absence of any commercial or financial relationships that could be construed as a potential conflict of interest.

## Footnotes

^1^Though, of course, takeaway pizza, etc., is often served/eaten direct from the cardboard box in which it is delivered.

^2^The first shop selling fish and chips opened in 1860 in the East End of London (see also Spence, 2021c).

^3^Indeed, it has been suggested that the key asset Roofoods Ltd., Deliveroo's parent company, has is its consumer data (which, crucially, is not shared with restaurants; Walters, 2020).

^4^In the UK, the phrase “Deliveroo Editions” is key. It is code for the fact that your meal may actually have been prepared in a dark kitchen.

^5^Of course, in a non-food context, many top clothes brands fabricate their merchandise in sweatshops in the Third World, so perhaps the case of branded takeaway food is not so very different after all.

^6^For instance, the “Zillion Dollar Frittata” at Norma's in New York City, a decadent $2,000 egg dish including lobster and caviar is ordered by less than a dozen customers a year, but its inclusion on the menu is meant to evoke a feeling of extravagance and playfulness (Haviland-Blunk, bib48).

^7^It has even been suggested that the quality of the bathroom fittings might play a role in the Michelin guides rankings (Sharp, 2013). As Steinberger (2010, p. 75) put it: “*The Michelin Man, they came to believe, had a yen for luxury and wanted his surroundings to be as sumptuous as his food*.” Not too far removed from this suggestion is the claim that one should never eat at a restaurant where the facilities are of questionable cleanliness (see Markwell, 2017).

^8^Dickins and Schalz (2020) have even put forward an explanation couched in terms of evolutionary theories of foraging, to explain the panic buying (e.g., of foods such as pasta).

^9^Note here also how trends in aesthetic preference are cyclical (Carbon, 2010, 2011).

^10^Indeed, guests are sent home from Vespertine with a small bottle of the restaurant's bespoke scent.

^11^As Scattergood (2017) puts it: “Kahn's dishes, though expertly orchestrated from seasonal ingredients, are sometimes indistinguishable from the ceramics they're presented with, resembling not so much food as *objets d'art*.”

^12^Notice also how the format of the bento box is perfectly adapted to remote consumption, with all of the elements served at room temperature, and tightly segmented in the sections of a molded tray, meaning that contents stay in place even when bento box is carried around.

^13^Indeed, the question has been raised in the press as to whether dark kitchens will do to independent restaurants what Amazon has already done to small local shops in UK and North America?

^14^Note also here how pre-Covid a number of restaurants were already targeting their offering to the solo diner (see Spence, 2017a).

^15^This, note, a version of the in-restaurant personalization that the restaurant is so famous for.

^16^Mukbang can perhaps be considered one of the solutions to digital commensality which has become surprisingly popular in the Far East over the last 5 years or so (e.g., in Korea; see Pereira et al., 2019).

^17^Note here that there is a whole line of gastrophysics research to be conducted around ascertaining the minimum conditions necessary to make the diner/consumer feel that they have contributed substantively to the creation, or finishing, of a dish (without their having to work too hard). Though, see also the long history on the role of adding fresh eggs to the powdered cake mix, which was supposed to be an essential part of making the North American homemaker really feel that they were making something for her family with love (Park, 2013).

^18^Roast chicken presumably an especially good choice as it may count as a comfort food (Spence, 2017b; see also Coppin, 2020).

^19^There is a sense in which this starts to come close to the immensely-popular Chef Steps (https://www.chefsteps.com/), or other chef-teaching apps or experiences, such as Masterclass (see also Peters, 2020; Pigott, 2020).

^20^Though, as of 15^th^ March, 2021, the website states only that the restaurant is currently closed.

^21^If you were wondering about the Selenite Charging Crystal, according to the accompanying text: “*The gemstone of purification: This variety of gypsum stone can be amazing for shifting through energy blocks, bringing peace and purity to one's head and heart, and generally just ensuring that you stay protected and connected to the world around you.”* So now you know!

^22^It has been suggested that this was, in part, in response to stiff competition from burger franchises, such as Five Guys (Barnes, 2017).

^23^While the format (CD) seems quaintly outdated nowadays, the emergence of curated playlists to match the food/drink is a rapidly expanding enterprise (Spence, 2019, 2020d).
